# Ovarian cancer: emerging concept on cancer stem cells

**DOI:** 10.1186/1757-2215-1-4

**Published:** 2008-10-12

**Authors:** Moorthy P Ponnusamy, Surinder K Batra

**Affiliations:** 1 Department of Biochemistry and Molecular Biology, University of Nebraska Medical Center, Omaha, NE 68198-5870, USA; 2 Eppley Institute for Research in Cancer and Allied Diseases, University of Nebraska Medical Center, Omaha, NE 68198-5870, USA

## Abstract

Emerging evidence suggests that the capacity of a tumor to grow and propagate is dependent on a small subset of cells within a tumor, termed cancer stem cells. In fact, cancer cells, like stem cells, can proliferate indefinitely through a dysregulated cellular self-renewal capacity. Cancer stem cells may originate due to the distribution into self-renewal and differentiation pathways occurring in multi-potential stem cells, tissue-specific stem cells, progenitor cells and cancer cells. Recent studies have shown that ovarian cancer also contains stem cells or tumor-initiating cells. Moreover, ovarian serous adenocarcinomas were disaggregated and subjected to growth conditions to select for self-renewing, non-adherent spheroids previously shown to be derived from tissue stem cells. A recent study showed that epithelial ovarian cancer was derived from a sub population of CD44^+^, CD117^+ ^and CD133^+ ^cells. The existence of cancer stem cells would explain why only a small minority of cancer cells is capable of extensive proliferation of the tumor. In this review, we have discussed the studies on ovarian cancer stem cells along with the molecular pathways that could be involved in these cancer stem cells.

## Introduction

Ovarian cancer is the fifth leading cause of cancer deaths and has the highest mortality rate among gynecologic cancers. It is the most lethal malignancy of the female reproductive system, at the initial stage the five-year survival rate is nearly 45%, which declines to 30% for patients with an advanced disease [1,2]. Greater than 90% of ovarian cancers arise from the surface epithelium [3], and tumorigenesis has been associated with ovulation-associated wound repair and/or inflammation, possibly leading to abnormal stem cell expansion [3,4]. Over the last several years, it has been increasingly evident that a small population (less than 5%) of cancer cells, referred to as "**cancer stem cells (CSCs)**", is responsible for the aggressiveness of the disease, metastasis and resistance to therapy [5-7]. Cancer stem cells, like somatic stem cells, are thought to be capable of self-renewal or unlimited proliferation [7]. The recent discovery that CSCs express certain 'stem cell-specific' markers has renewed interest and provided a rise in the idea that CSCs may arise from somatic stem/progenitor cells. Considerable research efforts have been directed toward the identification of cancer stem cell markers in ovarian cancer.

Stem cells, as classically defined, are cells with a capacity for self-renewal and generation of daughter cells that can differentiate into all the way down different cell lineages found in the mature tissue [8]. Stem cells always undergo asymmetric cell divisions, with each cell generating two cells; one that is identical to itself in stemness and another which is committed to a certain lineage. The daughter cell with stem cell like properties maintains its own compartment over time, while its sister cell undergoes a series of cell divisions [9]. Self-renewal allows stem cells to persist during the entire the lifetime of the organism, while their differentiation potential allows them to perform functions like tissue genesis, tissue maintenance, and regeneration following stress or injury [9].

Of all the types of stem cell, hematopoietic stem cells (HSCs) are the best characterized adult stem cell [10]. HSCs can differentiate to form mature blood cells but can also reproduce themselves, which is known as self-renewal [10]. It is reside in distinct stem-cell niches that vary in location depending on the developmental stages of organism [11]. The human HSCs express high level of CD34 and low or absent level of CD33, CD38, thy-1, and CD71, appears to be enriched for primitive progenitor and HSC activity, while more mature progenitors express one or more of these markers [12]. Furthermore, in therapeutic target hematopoietic stem cells are the only stem cells developed up to therapy for the cancer and other disorders for the blood [11] and following HSC study for other stem cells will lead to improve therapy for other cancers.

**Figure 1:**
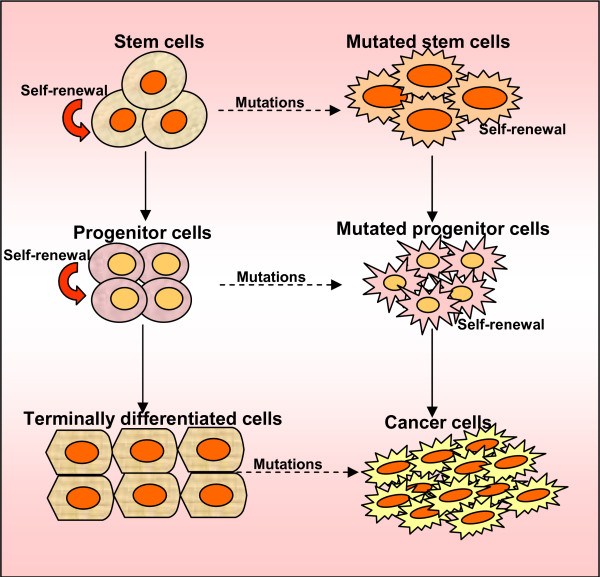
**Origin of cancer stem cells. Self-renewal and differentiation potentials are the features of stem cells**. Progenitor cells, the product of stem cells that lose the activity of self-renewal, could differentiate into mature cells, which have the feature of differentiation. The hypothesis is that cancer stem cells are caused by transforming mutations occurring in multi-potential stem cells, tissue-specific stem cells, progenitor cells, mature cells, and cancer cells.

Cancer stem cells may arise following transforming mutations that occur in untransformed stem cells, progenitor cells, mature cells, and cancer cells. The genetic program controlling self-renewal and differentiation plays a key role in the genesis of cancer stem cells (Figure 1). Cancer stem cells (CSCs) have been demonstrated to have roles in several cancers, including cancers of the ovaries, breast, brain, prostate, pancreatic, hepatocellular, head and neck cancers and hematological malignancies [5-7,13-27]. According to the CSC model, only a specific subset of the cancer cell population (i.e., the long-lived CSC subset) should be able to sustain *in vivo *tumor growth, whereas all other subsets (i.e., the tumor counterparts of short-lived differentiated cells) should not. Indeed, this assumption has now been repeatedly confirmed in several tumor systems. Three key observations classically define the existence of a CSC population: (i) Only the minority of cancer cells within each tumor are usually endowed with tumorigenic potential when transplanted into immunodeficient mice; (ii) Tumorigenic cancer cells are characterized by a distinctive profile of surface markers and can be differentially and reproducibly isolated from non-tumorigenic ones by flow cytometry or other immunoselection procedures; and (iii) Tumors grown from tumorigenic cells contain mixed populations of tumorigenic and non-tumorigenic cancer cells, thus recreating the full phenotypic heterogeneity of the parent tumor [28]. Furthermore, recent studies have been shown the functions of normal and malignant stem/progenitor cells in tissue regeneration, cancer progression and targeting therapies [29,30]. In this review we aim to provide insight into the evaluation of the evidence that supports the existence of cancer stem cells and the characterization studies that have tried to identify ovarian cancer stem cells. We also discuss how taking this subpopulation of cells into account may affect the way we treat ovarian cancers in the future.

### Cancer stem cells

The identification of a reservoir of stem cells within many adult tissues raises the interesting possibility that all adult tissues have stem cells. Stem cell populations within normal tissues are defined by certain common characteristics: self-renewal to maintain the stem cell pool over time; regulation of stem cell number through a strict balance between cell proliferation, cell differentiation and cell death; and the ability to give rise to a broad range of differentiated cells [31,32]. It is observed that like stem cells, cancer cells are widely thought to be able to proliferate indefinitely through a deregulated self-renewal capacity. In fact, cancer stem cells can thus only be defined experimentally by their ability to generate continuously growing tumors. CSCs have the capacity to self-renew, undergoing divisions that allow the generation of more CSCs and ultimately some of them differentiate into the various cell types that compose the tumor mass. To date, the practical translation of this definition, and the gold standard to define the 'stemness' of cancer cells, has been their ability to generate a phenocopy of the original malignancy in immuno-compromised mice [7].

### Evidence for the existence of cancer stem cells

To assay the cancer stem cells, a xenograft model for breast cancer was developed that allowed specific cancer tumors isolated directly from a patient to be passaged reliably *in vivo*. In this model, only a subset of cancer cells had the ability to form new tumors [5]. The cancer stem cells isolated from tumors are mostly isolated by flow cytometry as the CD44+ CD24-/^low ^lineage cell population [5]. Furthermore, dilution assays demonstrated that as few as 100 tumorigenic cancer cells were able to form tumors, while tens of thousands of the other (non-CSCs) populations of cancer cells failed to form tumors in nude mice. These tumorigenic cells have been serially generated in new tumors containing additional CD44+ CD24-/low lineage tumorigenic cells as well as the phenotypically mixed population of non-tumorigenic cancer cells [5,7]. In addition, when cultured cells were isolated based on the expression of CD133, a marker expressed by normal CNS stem cells [33], only the CD133+ fraction of cells was capable of forming spheres. These studies suggest that CNS tumors of neural origin contain a stem cell population. Li *et al*. reported that a highly tumorigenic subpopulation of pancreatic cancer cells expresses the cell surface markers CD44, CD24 and epithelial-specific antigen (ESA) [18]. Table 1 summarizes the studies which have described the direct isolation of populations containing cancer stem cells in various malignancies. Another phenotype used to distinguish these cells is their presence within the Side Population fraction as determined by their ability to exclude the Hoechst dye [34].

**Table 1:** Cancer type and specific marker for cancer stem cell populations

S. No	Cancer type	Markers for CSC population	References
1.	Brain Tumors	CD133^+^	[23]
2.	Breast Cancer	CD24^-/low^/CD44^+^/ESA^+^	[5]
3.	Ovarian Cancer	CD133^+^/Side population (SP)/CD44^+^, CD117^+^	[25,27,59]
4.	Lung Cancer	CD133^+^	[15]
5.	Prostate Cancer	CD44^+^/α2β1^high^/CD133^+^	[14]
6.	Pancreatic Cancer	CD44^+^/CD24^+^/ESA/CD133^+^	[16,18]
7.	Hepatocellular Cancer	CD133^+^	[24,26]
8.	Hematological Malignancies	CD34^+^/CD38^-^	[17]
9.	Colon Cancer	CD133^+^/CD44^+^/Lin^-^/ESA^+^	[22,28,44]
10.	Head and Neck Cancer	CD44^+^	[21]

### Therapeutic targets for cancer stem cells

The field of stem cell research has given new hope for the treatment and even a cure for incurable diseases in human. Particularly, the identification of a rare population of adult stem cells in most tissues/organs in humans has emerged as an attractive source of multiple stem/progenitor cells for cell replacement-based therapies and tissue engineering in regenerative medicine. Our recent review discussed that cancer stem/progenitor cell research also offers the possibility of targeting these undifferentiated and malignant cells that provide critical function in cancer initiation and relapse for treating patients diagnosed with advanced and metastatic cancer [30,35,36]. Various strategies consisting of molecular targeting of distinct oncogenic signaling elements activated in the cancer progenitor cells and their local microenvironment during cancer progression can be explored [37]. Furthermore, overcoming the intrinsic and acquired resistance of cancer stem/progenitor cells to current clinical treatments represents a major challenge in treating and curing the most aggressive and metastatic cancers [38]. In addition, hematopoitic stem cells are the most characterized stem cells and it has been used for the therapy to cure cancer [11]. In this review we also described that the molecular mechanisms involved in the intrinsic and acquired resistance of cancer cells to current cancer therapies [38].

### Pathways of self-renewal and carcinogenesis

**Figure 2:**
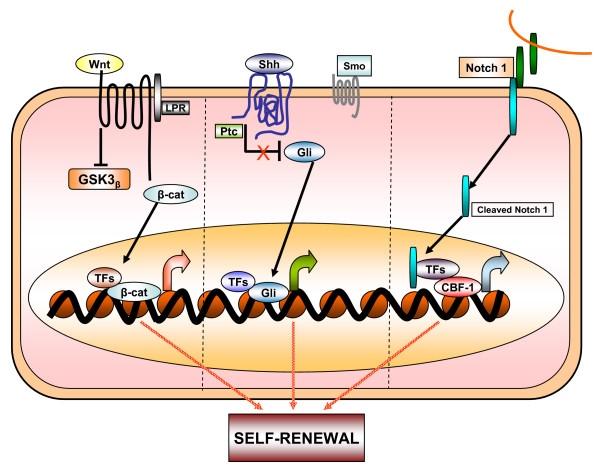
**Schematic diagram of signaling pathways that are involved in normal and cancer stem cell biology**. Wnt, Shh and Notch1 pathways have been shown to contribute to the self-renewal of stem cells and/or progenitors in a variety of organs, including the ovarian system. When deregulated, these pathways can contribute to oncogenesis. Mutations of these pathways have been associated with a number of carcinomas.

Since the cancer stem cells share common properties with normal stem cells, it is reasonable to think that they have overlapping regulatory mechanisms. Indeed, one of the most outstanding questions concerning the biology of stem cells is: how do multi-potent stem cells select a particular differentiation pathway and start to differentiate? Another question is how do stem cells decide to maintain self-renewal properties and continue to proliferate? Recent studies demonstrate that the presence of various genes and signaling pathways are involved in the regulation of the aforementioned processes. Among these, the Sonic Hedgehog (Shh), Notch and Wnt signaling transduction pathways play a major role in the self-renewal of stem cells [39-41]. Recent advances in the understanding of the role of Wnt, Hedgehog, Shh, and Notch signaling pathways in regulating stem cell self-renewal have shed new light on carcinogenesis (Figure 2) [7,42,43]. The next obvious question is the possible connection between tumors and the (Hedgehog) Hh and Wnt pathways and how the activation of these pathways leads, in some cases, to such highly efficient tumorigenesis. Recent genetic evidence suggests that somatic stem cells are the producers of CSCs; that the Wnt and Hh pathways function in the normal regulation of stem-cell number in at least some tissues; and that expansion of the somatic stem-cell population may be the first step in the formation of at least some types of cancers [44-46]. Numerous arguments support a stem-cell origin for human cancer. Foremost is the observation that stem cells possess many of the features that characterize the malignant phenotype, including self-renewal and unlimited replicative potential [47]. Also, the mutations that initiate tumor formation seem to accumulate in cells that persist throughout life, as suggested by the exponential increase of cancer incidence with age. This is thought to reflect a requirement for four to seven mutations in a single cell to effect malignant transformation [47]. Although similar signaling pathways may regulate self-renewal in normal stem cells and cancer stem cells, there are mechanistic differences in some cancers. Interestingly, the mechanistic differences in self-renewal between normal stem cells and cancer stem cells can thus be targeted to deplete cancer stem cells without damaging normal stem cells.

### Ovarian tumors

**Figure 3:**
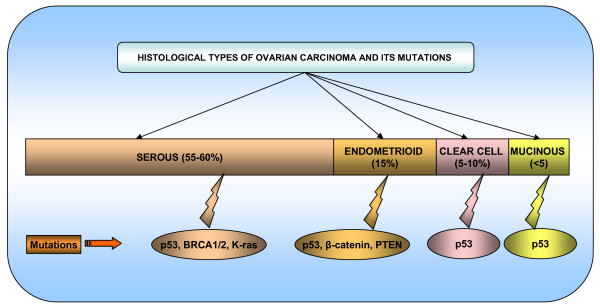
**Schematic diagram representing the histological types and its specific mutations in ovarian carcinoma**.

The ovaries contain three main types of cells germ cells, stromal cells and epithelial cells which give rise to germ cell, stromal and epithelial ovarian tumors, respectively. Epithelial ovarian cancers (EOC) were the most common type of ovarian cancers. Comprising nearly 90% of all ovarian cancers EOCs are derived from relatively pluripotent cells of the celomic epithelium or "modified mesothelium". These cells originate from the primitive mesoderm and can undergo metaplasia. Approximately 10% to 20% of epithelial ovarian neoplasms are borderline or low malignant potential tumors and are characterized by a high degree of cellular proliferation in the absence of stromal invasion. Of the invasive epithelial ovarian cancers, about 55–60% are serous, 15% endometrioid, 5–10% clear cell and <5% mucinous [48] (Figure 3). The various histological subtypes of ovarian carcinoma have identifiable precursor lesions and multiple early genetic alterations. Figure 3 explains the various histological subtypes of ovarian cancer and their associated specific mutations. Mutations may be one of the major factors contributing to the origin of ovarian cancer stem cells. Many of the histological subtypes resemble the epithelial component of the lower genital tract, including papillary serous tumors that have an appearance resembling the glandular epithelium lining the fallopian tube. Mucinous tumors, on the other hand, contain cells resembling endocervical glands, and endometrioid tumors contain cells resembling the endometrium. Non-epithelial types of ovarian cancer include sex cord-stromal tumors (6% of ovarian cancers) and germ cell tumors (3% of all malignant ovarian neoplasms) [49-51]. The histological subtypes of ovarian carcinoma have identifiable precursor lesions and early genetic alterations. Figure 3 explains the histological subtypes and its specific mutations in ovarian carcinoma. Mutations are one of the major alteration factors for the origin of cancer ovarian stem cells.

### Markers and their roles in ovarian tumors

In general, tumor markers can be used for one of four purposes: (i) screening a healthy population or a high risk population for the presence of cancer; (ii) making a diagnosis of cancer or of a specific type of cancer; (iii) determining the prognosis of a patient; and (iv) monitoring the course in a patient in remission or while receiving surgery, radiation, or chemotherapy.

Furthermore, recent studies have identified different prognostic and diagnostic surface markers for ovarian cancer [52] and these markers need to be analyzed for their role in ovarian cancer. One of the well-known tumor antigens is the epithelial cell mucin MUC1, a transmembrane glycoprotein that is differentially expressed on tumor cells compared with normal epithelial cells [53,54]. MUC1 is expressed either not at all or in small amounts on various normal epithelia but aberrantly or neoexpressed at high levels on the majority of adenocarcinomas. Tumor-associated alterations of MUC1 are characterized by hypoglycosylation, increased sialylation, and altered carbohydrate core-type expression [53]. Engelmann *et al *reported that MUC1 molecule is not only expressed on mature cancer cells, but also on tumor cells that have multiple characteristics of stem and progenitor cells [55]. This study demonstrates MUC1 expressed breast cancer cell line MCF7 as a source of a minor population of cells with characteristics of tumor stem/progenitor cells to show for the first time that these cells also express the hypoglycosylated (tumor) form of MUC1, previously described only on mature MCF7 cells and other tumors and tumor cell lines. Moreover, these cells give rise to MUC1^+ ^tumors *in vivo *and that these tumors maintain a small population of MUC1^+ ^cells with the stem/progenitor characteristics [55]. Our recent finding demonstrated the tumor-specific expression of Tumor Associated Glycoprotein-72 (TAG-72) in ovarian cancer and its association with disease stage may serve as a potential marker for effective disease management [56]. In addition, surface marker mucins are overexpressed in many epithelial malignancies including ovarian cancer, suggesting a possible role in the pathogenesis of these cancers. Other studies from our laboratory have provided experimental evidence that the MUC4 mucin interacts with HER2 potentiates its downstream signaling and enhances the motility of ovarian cancer cells. Our findings provide experimental support for the hypothesis that MUC4 mucin expression is associated with a higher metastatic potential and thereby a poor prognosis in ovarian cancer [57]. The future direction of these studies will be to explore the roles of MUC4 and TAG-72 in ovarian cancer stem/progenitor cells.

### Ovarian cancer stem cells

A recent study describes that ovarian cancer cell lines were shown to possess "side population" (SP) cells that have been described as cancer stem cells due to their stem-like characteristics including the ability to differentiate into tumors with different histologies. These putative cancer stem cells reflect the various histological subtypes observed in ovarian carcinoma. They also provide a model of cancer metastasis in which these cells are able to colonize, expand, and differentiate into heterogeneous tumor phenotypes similar to primary tumors. In such a model, both the primary tumors and metastasis would display similar genetic and expression profiles because both populations are supposedly derived from the same lineage of cancer stem cells [58]. Ovarian cancer stem cells, like somatic stem cells, are shown to be capable of unlimited self-renewal and proliferation. In general, multi-potent cancer stem cells may account for the histological heterogeneity often found in tumors [25,27,59]. Moreover, ovarian somatic stem cells would be expected to divide asymmetrically, yielding both a daughter cell that proceeds to terminal differentiation, and an undifferentiated copy capable of self-renewal. Repeated asymmetric self-renewal sets of somatic stem cells or their immediate progenitor's stem cells lead to the accrual of mutations over time, which might ultimately lead to their transformation into cancer stem cells and malignant progression.

Furthermore, another study describes that two mouse ovarian cancer cell lines such as MOVCAR7 and 4306 contain candidate cancer stem cells [25]. These two murine ovarian cancer cells have large SP, making them suitable to study ovarian cancer stem cell biology. A similar, albeit very small, SP was also identified in the human ovarian cancer stem cell lines IGROV-1, SKOV-3 and OVCAR-3 and also in cells claimed from patient ascetic fluid [25]. Further, a study proved that isolated and characterized ovarian cancer-initiating cells (OCICs) are fully capable of reestablishing their original tumor hierarchy *in vivo*. These cells are very organized self-renewing, anchorage-independent spheres and were reproducibly dividable using antibodies against both CD44 and CD117 [27]. These OCICs were capable of intraperitoneal tumorigenesis and could serially propagate tumors in animals. Consequently, this study fulfills all currently accepted criteria for the existence of a subpopulation of tumor-initiating cells [27], and their specific detection and targeting could be highly valuable for therapy of recurrent, chemo-resistant disease. Whereas advanced ovarian cancer is generally initially responsive to standard chemotherapies (ciaplatin and paclitaxel), that responsive almost inevitably followed by drug resistant phenotype [2,60]. One accepted hypothesis about chemoresistance is standard therapies failed to target tumor progenitors, which are have like normal stem cells because of expression of membrane efflux transporters. Zhang et al showed that OCICs, under stem cell-selective conditions, over express ABCG2 and are more resistant to cisplatin and paclitaxel, suggesting a possible role for these cells in ovarian cancer chemoresistance [27].

## Conclusion and perspective

The aforementioned studies showed that a so-called ovarian cancer stem cell, with high-proliferative capacity, self-renewal properties and multi-lineage potential, could be responsible for tumor development and the differentiation of more mature epithelial ovarian cells contributing to tumorigenesis. There are important consequences for cancer treatment if the growth of tumors is at least in part, dependent on a cancer stem cell population. The cancer stem cell hypothesis posits that cancer stem cells are a minor population of self-renewing cancer cells that fuel tumor growth and remain in patients after conventional therapy has been completed. The hypothesis predicts that effective tumor eradication will require obtaining agents that can target cancer stem cells while sparing normal stem cells. Experimental evidence suggests that ovarian cancer stem cells are relatively resistant to conventional chemotherapeutic agents. Current cancer therapies often engender severe toxicity because of their general effects on all rapidly dividing cells. Identification of candidate targets for more specific mechanism-based cancer therapy using techniques such as gene chips could reveal signature patterns of transcriptional output which are characteristic of activated self-renewal pathways.

Emerging evidence suggests that these pathways also control patterning and growth in self-renewing adult tissues by regulating the stem-cell compartment. Thus, pharmacological inhibition of these pathways in the worst case might result in severe toxicity due to a loss of normal stem-cell compartments. Further research will be needed to determine whether continuous pathway activity is required in normal and tumor tissues, and whether these requirements differ sufficiently as to allow therapeutic intervention. Even if pathway inhibition is prohibited by normal physiological requirements, other mechanism-based approaches that exploit aberrant pathway activation might be feasible. It has been proposed that malignancy is determined in all tissues by mis-regulation of a common set of genes that control growth by affecting cell proliferation, apoptosis, invasion and angiogenesis. This hypothesis is supported by the demonstration that multiple types of normal human cells can be made tumorigenic by the expression of a defined set of viral and cellular proteins. Therapeutic agents for the treatment of such tumors might target not only self-renewal pathway components, but also other critical transcriptional targets of the self-renewal pathways, or proteins that co-operate with them to deregulate growth.

It is important that agents directed against cancer stem cells discriminate between cancer stem cells and normal stem cells. This will require the identification of realistic drug targets unique to cancer stem cells. The identification of such targets and the development of anti-cancer agents will require a deeper understanding of normal stem cell biology as well as cancer biology. More importantly, identification of the ovarian cancer stem cell would provide a critical step in advancing the development of novel therapeutic strategies in the management of ovarian cancer. Furthermore, characterizations of such progenitor or cancer stem cells in drug resistant (Ciaplatin, Paclitaxel and etc) manner for ovarian cancer will likely lead to a greater understanding of early events leading to the genesis of this elusive disease, in addition to providing new therapeutics targets aimed at the cells directly responsible for its propagation.

## Abbreviations

CSC: Cancer Stem Cell; CNS: Central Nervous System; ESA: Epithelial-Specific Antigen; Shh: Sonic Hedgehog; Hh: Hedgehog; EOC: Epithelial ovarian Cancer; OCIC: Ovarian Cancer Initiating Cells; SP: Side Population

## Competing interests

The authors declare that they have no competing interests.

## Authors' contributions

PPM participated in drafting the full manuscript and creating figures. SKB participated in substantial contribution to conception and revising it critically for important intellectual content.
